# Isolation, culture and characterization of *Arsenophonus* symbionts from two insect species reveal loss of infectious transmission and extended host range

**DOI:** 10.3389/fmicb.2023.1089143

**Published:** 2023-02-01

**Authors:** Pol Nadal-Jimenez, Steven R. Parratt, Stefanos Siozios, Gregory D. D. Hurst

**Affiliations:** Institute of Infection, Veterinary and Ecological Sciences, University of Liverpool, Liverpool, United Kingdom

**Keywords:** symbiosis, evolution, reproductive parasitism, transmission mode, *Arsenophonus*

## Abstract

Vertically transmitted “Heritable” microbial symbionts represent an important component of the biology and ecology of invertebrates. These symbioses evolved originally from ones where infection/acquisition processes occurred within the environment (horizontal transmission). However, the pattern of evolution that follows transition from horizontal to vertical transmission is commonly obscured by the distant relationship between microbes with differing transmission modes. In contrast, the genus *Arsenophonus* provides an opportunity to investigate these processes with clarity, as it includes members that are obligate vertically transmitted symbionts, facultative vertically transmitted symbionts, strains with mixed modes of transmission and ones that are purely horizontally transmitted. Significantly, some of the strains are culturable and amenable to genetic analysis. We first report the isolation of *Arsenophonus nasoniae* strain *a*Pv into culture from the ectoparasitic wasp *Pachycrepoideus vindemmiae* and characterize the symbiosis. We demonstrate maternal vertical transmission and find no evidence for paternal inheritance, horizontal transmission or reproductive parasitism phenotypes. This leads us to conclude this strain, in contrast to related strains, is a facultative heritable symbiont which is likely to be beneficial. We then report the serendipitous discovery and onward culture of a strain of *Arsenophonus* (strain *a*Pb) from the blue butterfly, *Polyommatus bellargus.* This association extends the range of host species carrying *Arsenophonus nasoniae*/*Arsenophonus apicola* symbionts beyond the Hymenoptera for the first time. We perform basic metabolic analysis of the isolated strains using Biolog plates. This analysis indicates all strains utilize a restricted range of carbon sources, but these restrictions are particularly pronounced in the *A. nasoniae a*Pv strain that is solely vertically transmitted. Finally, we demonstrate the *Arsenophonus* sp. strain *a*Pb from the blue butterfly can infect *Galleria* waxworms, providing a model system for investigating the functional genetics of *Arsenophonus*-insect interactions. These results are consistent with a model of reduced metabolic competence in strains evolving under vertical transmission only. The data also broadens the range of host species infected with nasoniae/apicola clade strains beyond the Hymenoptera, and indicate the potential utility of the *Galleria* model for investigation of symbiosis mechanism.

## Introduction

Heritable symbionts—microbes that are vertically transmitted from parent to offspring–represent an important component of the biology of invertebrates (Hurst, 2017). These symbionts can provide services to the host in the form of essential nutrient supplementation (Douglas, 2009), natural enemy protection (Xie et al., 2010), and thermal adaptation (Corbin et al., 2017). Further, their capacity to block viral replication (Hedges et al., 2008; Teixeira et al., 2008; Gong et al., 2020) enable their use in pest and vector control, as a public health intervention interrupting the competence of arbovirus vectors (Nazni et al., 2019; Utarini et al., 2021). In some cases, they are obligately required by the host for development or reproduction, and in most cases the microbes are fastidious and unable to grow outside of a host environment. Contrastingly, heritable microbes can also act as reproductive parasites, causing sex-ratio distortion and cytoplasmic incompatibility (Hurst and Frost, 2015). The impact symbionts have on individual hosts–both beneficial and parasitic–cascades to ecological and evolutionary dynamics. Symbionts, for instance, drive changes in natural enemy-host dynamics (Ryder et al., 2014), alter patterns of sexual selection (Charlat et al., 2007), and potentiate host biodiversity over macroevolutionary timescales (Miller et al., 2021).

Vertically transmitted symbioses originally evolved from symbioses involving horizontal (infectious) transmission (Sachs et al., 2011), which either involved infection of a host by an environmental microbe, host capture of an environmental microbe, or an interplay between the parties (Hurst, 2017). Vertical transmission from parent to offspring would then have evolved as a means by which hosts retain useful symbionts, or as a mechanism for microbes to acquire new hosts (Sachs et al., 2011).

Investigating the processes that accompany transitions in symbiont transmission mode remains a key challenge, as existing vertically transmitted microbes are commonly evolutionary distant from horizontally transmitted species, obscuring the processes that occur early in the evolution of vertical transmission. In order to understand the processes that occur on first evolution into vertical transmission, we must compare symbionts with vertical transmission to closely related strains where symbiosis establishes horizontally through the environment. The contrasts then allow us to establish the tempo and mode of gene loss during vertically transmitted symbiosis, as well as allowing comparative analysis of the properties of different modes of symbiosis.

*Arsenophonus* is a microbial genus engaged in diverse symbiotic interactions with arthropod host species. Within the clade there are horizontally transmitted species (*A. apicola*) (Drew et al., 2021; Nadal-Jimenez et al., 2022), symbioses with mixed modes of transmission (*A. nasoniae* and insect-vectored *Canditatus* A. phytopathogenicus plant pathogens) (Bressan et al., 2011; Parratt et al., 2016), vertically transmitted symbionts that are not required for host function (e.g., *Cand.* A. triatominarum) (Hypsa and Dale, 1997), and obligately required vertically transmitted symbionts (e.g., *Cand.* A. arthropodicus and *Cand.* A. lipopteni from hippoboscid flies) (Dale et al., 2006; Nováková et al., 2016). The relationships vary from likely pathogenic (*A. apicola* in honey bees), through reproductive parasitism (*A. nasoniae* in *Nasonia* wasps), to mutualism (*Cand.* A. arthropodicus and *Cand.* A. lipopteni from hippoboscid flies). A key feature enabling functional analysis in this clade is the ability to isolate and grow some of the strains within this clade in cell-free culture, with *A. nasoniae* and *A. apicola* both amenable to culture and genetic manipulation (Nadal-Jimenez et al., 2019; Nadal-Jimenez et al., 2022). *Arsenophonus nasoniae* itself also represents an unusual microbe, possessing a very complex genome containing a high density of prophage elements and hyperdiverse extrachromosomal elements (Frost et al., 2020).

In this paper, we report the isolation and characterization of two strains of *Arsenophonus* from the *A. nasoniae*/*A. apicola* subclade that to date is dominated by strains which retain at least some infectious transmission. Previous work had reported *Arsenophonus* infection in *Pachycrepoideus vindemmiae*, an ectoparasitic wasp of fly pupae (Duron et al., 2010); we isolated this microbe to pure culture and characterized its phenotype in the wasp host in terms of transmission and reproductive parasitism. We then report the isolation of an *A. nasoniae*/*A. apicola* relative from the adonis blue butterfly, *Polyommatus bellargus* whose presence was detected in a population genomic study. We then compare their carbon utilization sources and inhibitory factors for these strains, using Biolog plates to investigate if strains that have transitioned to pure vertical transmission have reduced metabolic flexibility, and examining density obtained during *in vitro* culture. Finally, we report their capacity to grow in *Galleria melonella* waxworms, a model system for understanding the mechanistic basis of host-microbe interactions (Ménard et al., 2021).

## Materials and methods

### Isolation and characterization of *Arsenophonus* in *Pachycrepoideus vindemmiae*

The *P. vindemmiae* line used in these experiments was derived from a single *Arsenophonus*-infected female caught near Pierrefeu, southeast France (Duron et al., 2010). *Pachycrepoideus vindemmiae* is a *Drosophila* pupal parasitoid that lays a single egg in a fly host. Isolation of the symbiont was achieved by extracting wasp pupae from their drosophilid hosts, surface sterilizing them with 70% EtOH, washing with ddH_2_O and homogenizing in sterile PBS. Homogenate was then spread onto cell-free GC agar media (BD Difco, UK, 228950) supplemented with 3 ml/L isovitalex (BD Difco, UK, 211875) as described previously (Darby et al., 2010) and allowed to grow at 25^°^C for 4–6 days until single colonies were visible. A single clone was then picked into 100 μl of sterile PBS, 20 μl aliquots of which were spread onto fresh GC agar plates enriched with isovitalex using sterile resin beads to form bacterial lawns after a second bout of incubation and growth. Microbes were examined visually under a binocular microscope and compared to the colony morphology of the type species, *A. nasoniae* [mucoid, gray-white, round, and convex with entire edges and a “cauliflower” appearance– for image see (Nadal-Jimenez et al., 2019)]. Microbe identity was confirmed through colony PCR amplification of the 16S rRNA amplicon using primers 27F and U1492R (Turner et al., 1999), sequencing the amplicon using the original primers, end trimming the sequences manually by eye and retaining quality sequence which was then assembled in Geneious software v 6.1.8^1^ with the pair to establish a final 16S rRNA sequence. Best matches to this sequence on NCBI were then ascertained through a BLASTn search against the NCBI nr database using default parameters and top matches noted.

#### Estimating the vertical transmission of *Arsenophonus* in *P. vindemmiae*

To estimate the vertical transmission efficiency of *Arsenophonus* in *P. vindemmiae*, 20 infected female *P. vindemmiae* wasps were collected as pupae from stock vials, allowed to eclose and mate for 24 h with three infected males from their natal line. These females were then given *ad libitum* (100+) *D. melanogaster* (line Canton S, henceforth CS) pupae in which to oviposit individually for 48 h. After this time females were collected, DNA extracted using the Promega Wizard DNA extraction kit (Promega, UK, A1125), following standard protocols but with 1/4 volume of reagents to account for the small size of individual wasps. Infection status was then verified by PCR screening specific to *A. nasoniae* based on the 16S rRNA gene using the primers Arse16S-F and Arse16S-R (Arse16S–F: GGG TTG TAA AGT ACT TTC AGT CGT/Arse16S-R: CGC AGG CTC GCC TCT CTC, 30 reaction cycles: 15s 92^°^C melt, 59^°^C annealing 1^°^min, 30 s extension at 72^°^C) (Duron et al., 2008).

The adult progeny of infected females were collected after 30 days, allowing for full development and eclosion. Fifteen female progeny from each of the 20 infected mothers were then selected at random for PCR screening for *Arsenophonus* infection to generate a transmission efficiency value per mother. DNA was extracted as before, and DNA quality verified for each sample by amplifying a portion of the insect mitochondrial COI gene (Primers: LCO: 5′ GGT CAA CAA ATC ATA AAG ATA TTG G 3, HCO: 5′ TAA ACT TCA GGG TGA CCA AAA AAT CA 3′) (Folmer et al., 1994).

Infection prevalence under vertical transmission is reported as the observed proportion of offspring that tested positive for the symbiont, including 95% confidence intervals using the binom.confit interval function in R.

#### Capacity of *Arsenophonus* in *P. vindemmiae* to spread between lineages through superparasitism

Previous work reported horizontal transmission of *A. nasoniae* in another wasp, *N. vitripennis*, occurs when both infected and uninfected mothers share a host pupa. In *N. vitripennis*, vertical transmission is inefficient, and horizontal transmission is necessary for maintaining the symbiont infection (Parratt et al., 2016). To test whether *Arsenophonus* in *P. vindemmiae* also transmits horizontally between lineages of *P. vindemmiae*, we cohoused *Arsenophonus*-infected wasps with isogenic antibiotic-cured uninfected wasp females and scored infection prevalence in the emerging offspring.

The antibiotic cured line was obtained by rearing *P. vindemmiae* carrying *A. nasoniae* on *D. melanogaster* (CS) hosts that had been reared on ASG fly food containing 0.2% (w/v) rifampicin over two wasp generations. After this, forty mated females were isolated into separate isofemale lines for a further three generations on hosts that were not exposed to the antibiotic. At G5, females were reclaimed from their vials of hosts after 72 h of oviposition and screened for infection with *Arsenophonus* specific primers Arse16S-F and Arse16S-R and then established as an *Arsenophonus* negative line.

To test capacity for infectious transmission on superparasitism, we allowed 20 replicate sets of four female *P. vindemmiae* wasps to simultaneously co-lay in the same vial of *Drosophila melanogaster* pupae at a 2:2, *Arsenophonus* positive (*A+*): *Arsenophonus* negative ratio (*A*-) (we chose 2:2 rather than 1:1 to increase the chance of superparasitism events, which are inherently density dependent). Once females were grouped it was no longer possible to distinguish *A+* from *A*-. Therefore, following oviposition for 48 h, all mothers were collected and screened for *A. nasoniae* infection (80 total) to establish that two female wasps in the trials were infected with *A. nasoniae*; this screen resulted in three replicates being discarded because they had fewer than two infected females, giving a total of 17 replicates for analysis. We collected adult progeny that emerged from these cohoused lays after 30 days and PCR tested a random sample of 30 female offspring for *A. nasoniae* following previous methods. Our null expectation under the assumption that *A. nasoniae* only transmits vertically and is neutral to host fitness is that 50% of offspring emerging from each replicate will be infected with *A. nasoniae.* A binomial test against the null hypothesis of 50% prevalence was conducted in R, with the probability calculated from a one-sample Z-statistic.

#### Presence of maternal vs. paternal inheritance, sex ratio distortion and incompatibility phenotypes for *A. nasoniae a*Pv

*A. nasoniae* is as a sex-ratio distorter in the parasitoid *Nasonia vitripennis*, whilst other heritable symbionts distort sex ratios by inducing cytoplasmic incompatibility. To test for sex ratio distortion and cytoplasmic incompatibility in *P. vindemmiae—A. nasoniae a*Pv symbiosis, we performed a set of 2 × 2 factorial crosses of infected and uninfected male and female wasps. These crosses allow us to test for:

(i) Paternal and maternal transmission efficiency of *A. nasoniae a*Pv, which would be shown by *A. nasoniae a*Pv -infection in progeny of *A*- female x *A*+ male crosses and *A*+ female x *A*- male crosses, respectively.
(ii) Any sex ratio distortion phenotypes, which would be shown by differences in offspring sex ratios produced by *A+* females compared to *A-.*
(iii) Any cytoplasmic incompatibility, which would be shown if progeny from *A*- mothers crossed to *A+* fathers are either all male or have higher rates of failure to emerge as adults.

To this end, males and females for the crosses were collected within 24 h of eclosion and kept in mating groups of three males and three females according to treatment, with access to honey water for nutrition. We allowed mating in groups to reduce the impact of any infertile or unresponsive males in the experiment. After mating, females were removed and placed into individual “host vials” with 10 freshly pupated *D. melanogaster* (CS) (11 days old at 25^°^C) on removable plastic sticks embedded in their culture vials. Infection status of the parents was confirmed *post hoc* by PCR screening as described above. Final sample sizes of each cross after removal of replicates that died during oviposition or were not of the assigned infection status were as follows: 11 × both parents infected, 23 × infected fathers only, 16 × infected mothers only, 12 × neither parent infected. The number and sex ratio of the F1 offspring were scored for each clutch as they emerged. Where possible, up to two live F1 female offspring from each brood were taken for PCR screening for *A. nasoniae*. This was to determine whether transmission of the infection was maternal, paternal or biparental. The total number of offspring screened per treatment were: *N* = 12 both parents infected, *N* = 24 infected fathers, *N* = 16 infected mothers, *N* = 10 neither parent infected.

To test for differences in infection prevalence, sex ratio, and offspring viability between broods produced by crosses of differentially infected parents we used generalized linear models with binomial errors. In all cases the infection status of the parents was fitted as a categorical independent variable and the respective response variable as a binomial dependant variable. For infection prevalence, due to screening a maximum of two offspring per clutch, we grouped all individuals by treatment level prior to analysis. For brood viability and sex ratio we included a random intercept in our models to account for the nested nature of broods within treatments. Models were fitted with “lme4” in R and significance levels of main effects generated with type II Wald Chi square tests implemented by “car:Anova().” All probabilities and 95% confidence intervals were calculated with “binom:binom.confint()” using the logit method.

### Serendipitous isolation of an *Arsenophonus* sp. infecting the butterfly *Polyommatus bellargus*

As part of a separate population genomic study, thirty individual male *P. bellargus* were collected in August 2017 from Gloucestershire county, UK. Total DNA prepared from two legs from these specimens using the QIAamp DNA Micro Kit (Qiagen, UK). Sequencing libraries were prepared using the NEBNext Ultra II with 3 PCR cycles that was then sequenced on an Illumina HiSeq 4000 platform. Reads were trimmed and QCd using Sickle version 1.33^2^ and then checked for the presence of microbes and eukaryotic representation using Phyloflash (Gruber-Vodicka et al., 2020).

For the specimen indicating presence of *Arsenophonus*, remaining abdomen material for this specimen was retrieved from the −80^°^C freezer. Material was reconstituted in Brain Heart Infusion (BHI, Thermofisher, UK) and then grown onward on BHI agar plates at 30^°^C under standard aerobic conditions. Identity of the colony was confirmed as *Arsenophonus* through PCR assays combined with sequencing of 16S rRNA amplicons, as described above.

### Relatedness of strains

We estimated the relatedness of strains using the sequence of three marker genes: *fbaA* (encoding fructose bisphosphate aldolase class 2), *yaeT* (encoding an outer membrane protein common across gammaproteobacteria) and *ftsK* (encoding a core cell cycle gene) previously used to type *Arsenophonus* strains (Duron et al., 2010). Marker sequences for *Arsenophonus* sp. from *Polyommatus bellargus* were amplified using the original primers and conditions from Duron et al. (2010) (fbaA: fbaAf GCYGCYAAAGTTCRTTCTCC, fbaAr CCWGAACCDCCRTGGAAAACAAAA Tm=52°C Product=659bp; ftsK: ftsKf GTTGTYATGGTYGATGAATTTGC, ftsKr GCTCTTCATCACYATCAWAACC Tm=52°C product=445bp; yaeT: yaeTf GCATACGGTTCAGACGGGTTTG, yaeTr GCCGAAACGCCTTCAGAAAAG Tm=52°C product=473bp). Products were purified and Sanger sequenced with the original primers. Resulting chromatograms were manually checked for quality before being trimmed, assembled, and aligned against those of existing strains at protein level (see Supplementary Table 1) and then back-translated to nucleotide using the mafft aligner implementation in Geneious software v 6.1.8^1^. Alignment columns with more than 40% gaps were removed. Best fitting model estimations was performed using ModelFinder (Kalyaanamoorthy et al., 2017) as implemented in IQTREE (Nguyen et al., 2015). Phylogenetic relatedness of strains was estimated through Maximum Likelihood method, using IQTREE and the TN + F + G4 model with 1,000 ultrafast bootstrap replicates (Hoang et al., 2017).

### *In vitro* growth requirements of strains isolated

BIOLOG GEN III plates (Cat. No. 1030) were used to ascertain the *in vitro* physiological and biochemical characteristics of *A. nasoniae a*Pv from *P. vindemmiae* and *Arsenophonus* sp. strain *a*Pb from *P. bellargus* with comparison to those previously completed of *A. nasoniae a*Nv_FIN isolated from *N. vitripennis* collected in Marbury, UK, and *A. apicola* (CECT 30499*^T^*=DSM113403*^T^*=LMG 32504*^T^*) from *Apis mellifera* [both described in Nadal-Jimenez et al. (2022)]. All *Arsenophonus* strains were grown for 4–6 days (until a maximum OD_600_=0.4–0.6) in brain heart infusion (BHI) broth (Oxoid, UK) at 30^°^C and 250 rpm. The cultures were then spun down, and each pellet was resuspended in a tube of IF-A inoculating fluid (Biolog, Cat. No. 72401) and 100 μl of this suspension was added to each of the 96 wells of the Biolog GENIII plate. The plate was subsequently placed to incubate at 30^°^C without shaking for 6–8 days, to allow *Arsenophonus* growth and full development of the chromogenic medium before scoring in line with manufacturer’s instructions.

In addition, growth of *Arsenophonus* from *P. vindemmiae* and *Arsenophonus* from *P. bellargus* and *A. nasoniae* from *N. vitripennis* wasps [as described in Frost et al. (2020)] were compared in BHI media within the laboratory. 5 ml of BHI broth was inoculated with five microliters of stationary phase *Arsenophonus* in BHI broth (as ascertained through OD), and growth under atmospheric Oxygen monitored *via* colony forming units obtained on serial dilution plates, with 20 μl inoculum removed at intervals up to 72 h. Growth trajectories were 3-fold replicated and completed at 30^°^C and normal aerobic conditions.

### Capacity to grow in *Galleria*

*Arsenophonus* from *P. vindemmiae* and *Arsenophonus* from *P. bellargus* were independently transformed with plasmid pOM1:*:gfp* using previous protocols to establish green fluorescent protein (GFP) expressing clones. The capacity of these strains to infect was examined alongside GFP expressing *A. nasoniae* derived from *N. vitripennis* wasps (Nadal-Jimenez et al., 2019) and a GFP-expressing *E. coli* MG1655 harboring the same plasmid. In brief, strains were grown in BHI agar plates for 6 days (*A. nasoniae* and *Arsenophonus* from *Pachycrepoideus vindemmiae*); 2 days (*Arsenophonus* from *P. bellargus*) and overnight (*E. coli* MG1655), and then subcultured in BHI broth for two (*A. nasoniae* and *Arsenophonus* from *Pachycrepoideus vindemmiae*) and 1 days (*Arsenophonus* from *P. bellargus* and *E. coli*). 1 μl of culture (OD_600_=0.4–0.6) was injected into each of 25 individual second instar *G. mellonella* larvae using a nanoject III and remains of culture retained for estimation of bacterial density through serial dilution; in all cases, >10^5^ cfu were introduced. Sterile BHI media negative controls were also completed. Individual *G. mellonella* larvae were then monitored for visible infection on a binocular microscope under epifluorescence to excite GFP, scored as clear expression of GFP, over 3 days at x 25^°^C.

## Results

### Isolation and characterization of *A. nasoniae* from *Pachycrepoideus vindemmiae*

*A. nasoniae* was successfully isolated from *P. vindemmiae* to pure culture. Colonies were slow growing (5 days at 30^°^C), and morphology resembled previously characterized *A. nasoniae*. 16S rRNA sequence was obtained (Accession number OP289003) and had 100% identity at 16S rRNA to the type species *A. nasoniae* (Gherna et al., 1991). Thus, the isolate from *P. vindemmiae* can be regarded most parsimoniously as a strain within this described species. Adopting the nomenclature for other symbiont strains within microbial species, we refer to this strain henceforth as *A. nasoniae a*Pv (*Arsenophonus nasoniae* from *Pachycrepoideus vindemmiae*).

#### Estimating the vertical transmission of *A. nasoniae a*Pv in *P. vindemmiae*

**FIGURE 1:**
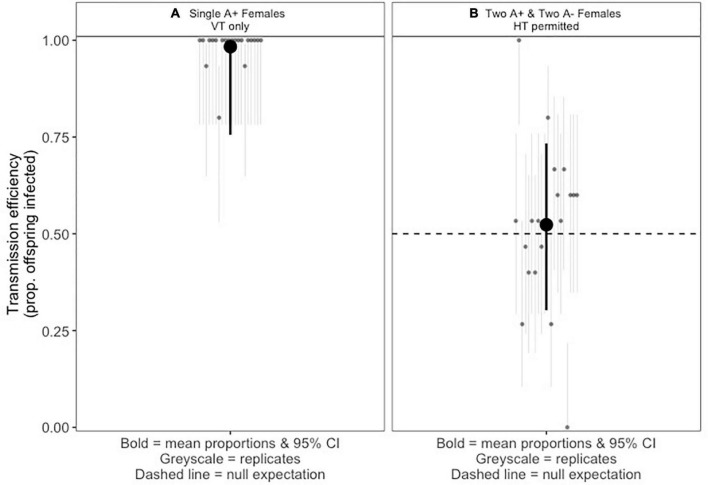
**(A)** Vertical transmission of *Arsenophonus nasoniae a*Pv in *Pachycrepoideus vindemmiae*. Light gray points and errors represent infection prevalence of 20 replicate broods laid by single infected mothers (15 randomly selected female offspring screened from each brood). Dark point and errors show the mean proportion of progeny infected and mean 95% confidence interval calculated using the logit method. **(B)** Fraction of *P. vindemmiae* progeny carrying *A. nasoniae a*Pv when half of mother wasps carry *a*Pv. Pale points and errors represent infection prevalence in 20 replicate broods co-laid by 2:2 infected:uninfected mothers (15 randomly selected female offspring screened from each brood). Dark points and errors represent mean infection prevalence and upper and lower 95% confidence intervals calculated with the logit method. Dashed line illustrates the null expectation of 50% infection if there is no horizontal transmission (HT) and no (dis)advantage to symbiont infection.

The *A. nasoniae a*Pv in its native host *P. vindemmiae* exhibited high vertical transmission efficiency. When a single infected female produces offspring, vertical transmission efficiency of *Arsenophonus* is estimated at 98.3% (Figure 1A).

#### Capacity of *A. nasoniae a*Pv to spread between in *P. vindemmiae* lineages through superparasitism

When two infected and two uninfected females share access to the same group of fly pupal hosts, the mean infection prevalence of wasp offspring emerging from the group is 53.3% (Figure 1B). This is not a significant deviation from the 50:50 infected/uninfected ratio we would expect to see for vertical transmission where half the female parents are infected (*P*=0.453, one-sample Z-test of proportions, confidence intervals: 0.465–0.581).

#### Presence of maternal vs. paternal inheritance, sex ratio distortion and incompatibility phenotypes for *A. nasoniae a*Pv

**FIGURE 2:**
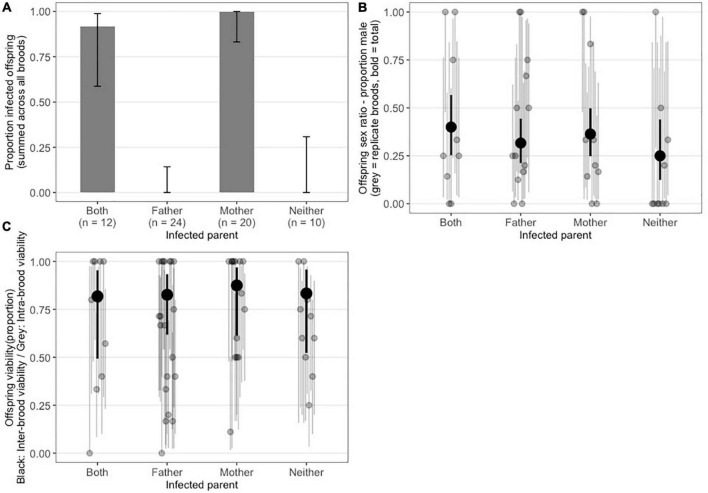
**(A)** Proportion of *P. vindemmiae* progeny positive for *A. nasoniae a*Pv partitioned by parental infection status: Both mother and father infected, father only, mother only, and neither. Results are given as the mean of replicate crosses, with error bars representing 95% binomial confidence intervals. **(B)** Offspring sex ratio (proportion male) from crosses with different infection status. Gray dots indicate individual families, Black dot represents mean across replicate families. **(C)** Offspring viability (proportion of pupae successfully parasitized with emerging wasp). Gray dots indicate individual families, Black dot represents mean across replicate families.

Our 2 × 2 cross design indicated vertical transmission was solely maternal. Infected progeny were observed only when the female parent was infected, with none of 24 offspring infected where the father was infected and the mother was not (95% Binomial Confidence intervals on paternal transmission rate: lower=0.0, upper=0.142) (Figure 2A). Complete separation of infection across treatment (i.e., no infection observed in broods with infected fathers only or in uninfected control broods) prevented statistical test of this differences. We also found no evidence for heterogeneity in clutch sex ratio between any of the treatments (Wald χ^2^=2.1, df=3, *P* = 0.55, mixed effect logistic regression model with replicates fitted as random intercepts) (Figure 2B). This is strong evidence against *A. nasoniae a*Pv causing CI or other sex ratio distortion phenotypes such as feminization or male-killing. Under CI, male-biased sex ratios are expected where the male partner was infected and the female partner uninfected (as male progeny in Hymenoptera are produced by arrhenotoky, they are not the product of sex and thus are not affected by CI) (Vavre et al., 2000). Other sex ratio distorting phenotypes should produce female-biased sex ratios when the female was infected irrespective of paternal infection status. Finally, we also find no significant differences in clutch viability between treatments (Wald χ^2^=0.23, df=3, *P*=0.97, Figure 2C). These data further indicate that infection with *Arsenophonus* does not cause incompatibilities between differentially infected parents and additionally indicate the symbiont does not enhance or reduce the probability of successful development of its wasp host.

### Isolation of *Arsenophonus* sp. from the butterfly *Polyommatus bellargus*

**FIGURE 3:**
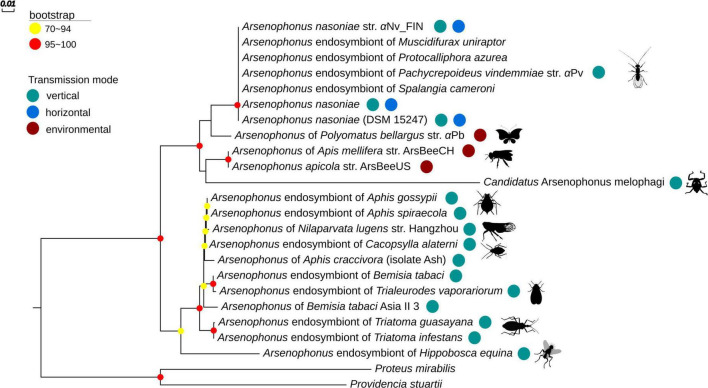
Estimation of *Arsenophonus* phylogeny using Maximum Likelihood (ML) based on the concatenated sequences of *fbaA, ftsK*, and *yaeT* genes [as in Duron et al. (2010)]. Only bootstrap values higher than 70% are shown (depicted by colored dots on nodes). The phylogenetic tree was constructed using IQTREE and the TN + F + G4 model. Transmission mode of *Arsenophonus* and stylized insect host are shown next to names. Accession numbers underpinning the phylogeny can be found on figshare (see data availability).

*Arsenophonus* sp. isolation was obtained from −80^°^C frozen material from the butterfly *Polyommatus bellargus* with growth in liquid culture and cloning to BHI agar achieved successfully. The strain was closely allied to *A. nasoniae/A. apicola* based on 16S rRNA sequence (OP203938). The relatedness of this strain to others was estimated based on concatenated *fbaA*, *ftsK*, and *yaeT* that we obtained (Accession numbers: OP205267-OP205269). This analysis indicated with high certainty that the strain lies within the *nasoniae*-*apicola* clade in which other culturable *Arsenophonus* lie. The strain is sister to other known *A. nasoniae* strains (Figure 3). Because affiliation with each of the formally described *Arsenophonus* species is uncertain on current data, we refer to this strain without specific affiliation as *Arsenophonus* sp. *a*Pb (from *Polyommatus bellargus*).

### *In vitro* growth requirements of strains isolated

We compared carbon utilization and inhibition across *A. nasoniae* isolated from UK *N. vitripennis* (*A. nasoniae a*Nv_FIN, mixed modes of transmission), from *P. vindemmiae* (*A. nasoniae a*Pv, vertical transmission) and *Arsenophonus* sp. *a*Pb from *P. bellargus*, with previously established growth conditions for *A. apicola* (horizontal transmission only). All strains utilized a narrower breadth of carbon sources than *A. apicola*, with the *a*Pv strain for *P. vindemmiae* having the narrowest range (Table 1). *A. nasoniae a*Nv_FIN from *Nasonia vitripennis* had the broadest capacity to thrive in the presence of inhibitory compounds. Growth of the *a*Pv strain was affected by a broad range inhibitory conditions and compounds, and growth of *a*Pb inhibited particularly by high NaCl and low pH (Table 2).

**TABLE 1:** Carbon utilization sources for diverse *Arsenophonus* strains as estimated from biolog plate analysis: *A. nasoniae a*Nv_FIN, *A. nasoniae a*Pv (from *P. vindemmiae*), *Arsenophonus* sp. strain *a*Pb (from *P. bellargus*).

	*A. nasoniae* strain aNv_FIN	*A. nasoniae* strain *a*Pv	*Arsenophonus* sp. strain *a*Pb	*Arsenophonus apicola* ArsBeeUS
D-glucose	+++	+++	+++	+++
D-mannose	+++	–	+++	+++
D-fructose	+++	+++	+++	+++
N-acetyl glucosamine	+++	+++	+++	+++
D-glucose-6-PO_4_	+++	+++	–	+++
D-fructose-6-PO_4_	+++	+++	–	+++
L-malic acid	+++	+++	–	+++
D-malic acid	–	–	–	+++
Bromo-succinic acid	+++	++	–	+++
D-gluconic acid	–	–	+++	+++
Glucoronamide	–	–	–	++
Methylpyruvate	++	–	+++	+++
L-aspartic acid	–	–	+++	+++
L-glutamic acid	++	–	+++	++
α-keto-glutaric-acid	++	–	–	+++
α-keto-butyric-acid	++	–	–	++
α-hydroxy-butyric-acid	–	–	–	++
Acetic acid	–	–	+++	–
L-serine	–	–	+++	–
Glycyl-L-proline	–	–	++	–
Acetoacetic acid	–	–	–	++

Data from *A. apicola* are given for comparison. −, no growth with this carbon source; +, very weak growth; ++, weak growth; +++, growth equivalent to full media. The following carbon sources were not utilized by any of the strains: Dextrin, D-maltose, D-Trehalose, D-Cellobiose, Gentiobiose, Sucrose, D-Turanose, Stachyose, D-raffinose, α-D-lactose, D-melibiose, B-Methyl-D-Glucoside, D-Salicin, N-Acetyl-B-D-Mannosamine, N-Acetyl-D-Galactosamine, N-Acetyl Neuraminic Acid, D-Galactose, 3-Methyl Glucose, D-Fucose, L-Fucose, L-Rhamnose, Inosine, D-sorbitol, D-mannitol, D-Arabitol, myo-inositol, Glycerol, D-Aspartic Acid, D-Serine, Gelatin, L-Alanine, L-Arginine. L-Histidine, L-Pyroglutamic Acid, Pectin, D-Galacturonic acid, L-Galactonic Acid Lactone, D-Glucuronic Acid, Mucic Acid, Quinic Acid, D-Saccharic Acid, *p*-hydroxy phenyl acetic acid, D-Lactic Acid Methyl Ester, Citric Acid, Tween-40, gamma-Amino-Butryric acid, B-Hydroxy-D-L-Butyric Acid, Propionic Acid, Formic Acid.

**TABLE 2:** Impact of environmental and xenobiotic stress conditions on growth of *Arsenophonus* strains on Biolog III plates.

	*A. nasoniae* strain aNv_FIN	*A. nasoniae* strain *a*Pv	*Arsenophonus* sp. strain *a*Pb	*Arsenophonus apicola* ArsBeeUS
pH 6	+++	++	+++	+++
pH 5	++	–	–	++
1% NaCl	+++	++	+++	+++
4% NaCl	+++	++	–	+++
8% NaCl	+++	++	–	+++
1% sodium lactate	+++	++	+++	+++
Fusidic acid	+++	++	+++	+++
D-Serine	+++	++	+++	+++
Troleandomycin	+++	++	+++	+++
Rifamycin SV	+++	++	+++	+++
Minocycline	+++	++	+++	+++
Lincomycin	+++	++	+++	+++
Guanidine HCl	+++	++	+++	+++
Niaproof 4	++	–	+++	–
Vancomycin	+++	++	+++	+++
Tetrazolium violet	++	++	+++	–
Tetrazolium blue	+++	++	+++	+++
Nalidixic acid	+++	++	+++	+++
Lithium chloride	+++	++	+++	+++
Potassium tellurite	+++	++	+++	+++
Aztreonam	+++	++	+++	+++
Sodium butyrate	+++	++	+++	+++
Sodium bromate	–	–	–	–

−, no growth; +, seriously inhibited growth; ++, inhibited growth; +++, no measurable inhibition.

Strains varied in their strength of aerobic growth in BHI media. All strains grew to equilibrium density in 72 h, with highest density obtained for *Arsenophonus* sp. *a*Pb (5.7 × 10^8^ cfu/μl), followed by *A. nasoniae* strain *a*Nv_FIN (7.2 × 10^7^ cfu/μl) with *A. nasoniae a*Pv showing two orders of magnitude lower growth (5.2 × 10^5^ cfu/μl).

### Capacity to grow in a *Galleria* infection model

**FIGURE 4:**
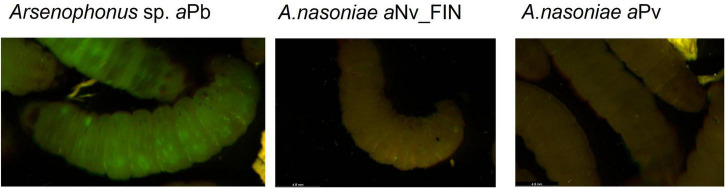
Exemplar images of *Galleria mellonella* larvae 48 h post-infection with *Arsenophonus* sp. *a*Pb, *A. nasoniae a*Nv_FIN, and *A. nasoniae a*Pv. Each *Arsenophonus* strain carries the plasmid pOM1::GFP, and larvae are viewed on a binocular microscope under epifluorescence, with green fluorescent protein (GFP) emitting green under excitation.

Neither *A. nasoniae a*Nv_FIN, nor *A. nasoniae a*Pv, established infection in the *Galleria* model, as measured through GFP fluorescence of injected larvae 2 and 3 days post-inoculation. In contrast, *Arsenophonus* sp. *a*Pb was capable of propagating in *G. mellonella* (Figure 4). Overall 22 of 25 injected individuals developed a disseminated infection when challenged with *Arsenophonus* sp. *a*Pb (Table 3). *Galleria mellonella* mortality was observed solely in 5 out of 25 larvae infected with the *E. coli* MG1655 control, and this was in the first 24 h post-injection. No mortality was observed over the 72 h period in any of experimental treatments.

**TABLE 3:** Alive/dead status of *G. mellonella* waxworm larvae, and presence of microbial proliferation as scored through GFP fluorescence status of the waxworm, over 3 days post-injection with various strains of *Arsenophonus*, with *E. coli* MG1655 known to be not capable of proliferation, and BHI medium injection as controls.

	Day 1			Day 2			Day 3		
	*Galleria* alive bacteria present	*Galleria* alive bacteria absent	*Galleria* dead	*Galleria* alive bacteria present	*Galleria* alive bacteria absent	*Galleria* dead	*Galleria* alive bacteria present	*Galleria* alive bacteria absent	*Galleria* dead
*A. nasoniae a*Nv_FIN	0	25	0	0	25	0	0	25	0
*A. nasoniae a*Pv	0	25	0	0	25	0	0	25	0
*Arsenophonus* sp. *a*Pb	20	5	0	23	2	0	23	2	0
*E. coli* MG1655	0	20	5	0	20	5	0	20	5
BHI control	0	20	0	0	20	0	0	20	0

*N*=25 for all cases except BHI control, *N*=20. *Galleria* alive bacteria present, waxworm alive with signs of bacterial proliferation as assessed by GFP fluorescence; *Galleria* alive bacteria absent, waxworm alive without signs of bacterial proliferation as assessed by GFP fluorescence; *Galleria* dead, waxworm had died.

## Discussion

The genus *Arsenophonus* comprises members that have horizontal (infectious) transmission (e.g., *A. apicola* in *Apis mellifera* honeybees) (Drew et al., 2021), reproductive son-killer parasites with mixed modes of transmission (*A. nasoniae a*Nv in *N. vitripennis* wasps) (Parratt et al., 2016), and a range of facultative and obligate vertically transmitted symbionts. In this paper, we examined the biology of two newly isolated *Arsenophonus* symbionts in the *apicola-nasoniae* group. We first ascertained the nature of the symbiosis between *A. nasoniae a*Pv and the ectoparasitic wasp *P. vindemmiae*. We then cultured a novel *Arsenophonus* from the butterfly *P. bellargus*, in this case a serendipitous isolation from a −80^°^C preserved butterfly abdomen. Finally, we compared the breadth of carbon utilization sources of the isolates to *A. nasoniae* and *A. apicola*, and capacity to infect *G. mellonella* waxworms.

*A. nasoniae a*Pv showed high (98%) maternal transmission fidelity in *P. vindemmiae* and no evidence of either paternal transmission or horizontal transmission. This observation contrasts to *A. nasoniae a*Nv in *N. vitripennis*, which relies on both vertical and horizontal transmission to persist in the parasitoid population (Parratt et al., 2016). The contrast with *A. nasoniae a*Nv makes sense in light of differences in the ecology of the host species*: N. vitripennis* is a gregarious parasitoid that commonly exhibits superparasitism in which two female wasps utilize the same fly pupa, providing an opportunity for horizontal transmission within the shared environment (Grillenberger et al., 2009). *Pachycrepoideus vindemmiae*, in contrast, lays a single egg per host fly, and rarely superparasites (Li et al., 2022). Thus, the difference in transmission modes in these *A. nasoniae* strains reflects in part distinct opportunities for horizontal transmission associated with differences in host biology.

The close relatedness of maternally inherited *A. nasoniae a*Pv to *A. nasoniae a*Nv which has mixed modes of transmission may therefore reflect a very recent transition of the *A. nasoniae a*Pv strain to a purely vertically transmitted lifestyle, though this conclusion awaits wider genomic analysis of the relatedness of the strains. Future research should examine if the mechanism of vertical transmission varies between *A. nasoniae a*Nv and *A. nasoniae a*Pv; the former is extracellular and transmitted in calyx fluid during wasp oviposition into fly pupae to be then taken up by F1 wasp larvae feeding on the fly pupa (Nadal-Jimenez et al., 2019). Whether *A. nasoniae a*Pv adopts this mode of vertical transmission (which has high rates of segregational loss) or has adapted to intracellular life for vertical transmission, warrants investigation.

The relationship between *A. nasoniae a*Pv and its wasp host is a facultative heritable symbiosis (uninfected hosts were generated through antibiotic treatment and are viable and fertile). Segregational loss during vertical transmission means the symbiont would be lost from populations in the absence of a “drive.” Our data rule out one form of drive–reproductive parasitism through either sex ratio distortion or cytoplasmic incompatibility– and in the absence of infectious transfer, these data imply a balancing benefit to infection. The nature of this benefit is uncertain; offensive and defensive symbiosis are possible, but the entomophagous nature of its *P. vindemmiae* host perhaps makes a nutritional role less likely.

Evolution of microbial symbionts that are vertically transmitted is typically reductive, with progressive loss of metabolic function associated with systems rendered redundant by permanent host association and the lack of need for active infection (McCutcheon and Moran, 2012). This process underpins the observation that the vast majority of heritable symbionts of insects are fastidious to culture. Our study highlights *A. nasoniae a*Pv as a strain that is maternally inherited and very closely related to *A. nasoniae a*Nv, that contrastingly has mixed modes of transmission in *N. vitripennis* wasps. Nevertheless, *A. nasoniae a*Pv retains the capacity for growth on cell-free media. Biolog analysis indicated this strain did have more restrictive requirements for culture, with a narrower range of carbon utilization sources, and growth was much weaker than for the other strains, achieving two orders of magnitude lower equilibrium density in culture under comparable conditions. Thus, this datum supports the conceptual view that metabolic competence reduces over evolutionary time when horizontal transmission is very rare. Onward, comparative genomic analysis of pathway loss in this strain will be instructive.

The finding of an *Arsenophonus* sp. infecting the butterfly *P. bellargus* extends the range of *A. nasoniae*/*A. apicola* clade symbionts beyond Hymenoptera for the first time. The infected individual was the only one carrying *Arsenophonus* in a sample of 30 male butterflies; the host died shortly after collection and had very high read depth of *Arsenophonus* redolent of those found during *A. apicola* infection. *Polyommatus bellargus* was the only eukaryotic signal present in the illumina reads, so we can be clear the *Arsenophonus* was associated with the butterfly and not a parasite or phoretic associate of the host. *A. apicola* exists as an environmentally acquired infection in the Hymenoptera pollinator community (Yanez et al., 2016; Drew et al., 2021), and it is tempting to suggest that *Arsenophonus* in this butterfly is likewise acquired *via* the pollinator environment through shared flower environments, and that it represents an opportunistic pathogen. Further experiments will be needed to assess this hypothesis. The recovery into culture of *Arsenophonus* sp. strain *a*Pb from this dead specimen supports a means for isolating further novel *Arsenophonus* strains through dividing hosts, testing one half by PCR assay whilst retaining the other half preserved at −80^°^C for onward culture in case the symbiont is detected.

Finally, our study indicates *Arsenophonus* sp. *a*Pb is capable of infecting *Galleria mellonella* waxworms, a model system for understanding host-microbe interactions (Kemp and Massey, 2007). This capacity contrasted with the two *A. nasoniae* strains. The capacity of the *Arsenophonus a*Pb strain to infect *Galleria* may reflect either the relatedness of the host species (both Lepidoptera), or a more generalist horizontal transmission network of *Arsenophonus* sp. strain *a*Pb in nature. The capacity of *Arsenophonus* sp. strain *a*Pb to grow in *Galleria* provides the opportunity for understanding the molecular basis of *Arsenophonus* interactions with insects, including interface with the host immune system. Unlike many other insect endosymbionts, *A. nasoniae* and *A. apicola* are predominantly extracellular symbionts/pathogens, exposed to humoral and cellular immune systems. The *Galleria* system will enable functional analysis of how *Arsenophonus* establishes infection in the face of these host responses.

## Data availability statement

The datasets presented in this study can be found in online repositories. Data underpinning Figures 1, 2 and onward analyses can be downloaded from https://doi.org/10.6084/m9.figshare.20628225.v1 and https://doi.org/10.6084/m9.figshare.20628798.v1. Code for statistical analysis can be downloaded at https://doi.org/10.6084/m9.figshare.21753653. The raw Illumina reads for the *Arsenophonus* infected *Polyommatus bellargus* butterfly have been deposited in Genbank under BioProject accession PRJNA911589.

## Author contributions

GH, SP, and PN-J: concieved the study. SS and GH: initial detection of strains. PN-J and SP: initial isolation of strains. SP: phenotypic assays in wasps and onward analyses. SS: phylogenetic analysis. PN-J: microbial bioassays. All authors contributed to the writing of manuscript and approved the submitted version.

## Footnotes

1
http://www.geneious.com/

2
https://github.com/najoshi/sickle
