# Supplementary material for: Isolation, culture and characterization of Arsenophonus symbionts from two insect species reveal loss of infectious transmission and extended host range
Source: Front Microbiol. 2023 Feb 1;14:1089143. doi: 10.3389/fmicb.2023.1089143 (PMC9928724; doi:10.3389/fmicb.2023.1089143)
Supplement: Supplementary file 1 [file Table_1.doc]

**Table S1.** Accession numbers of the sequences used for phylogenetic analysis.

| strain | fbaA | ftsK | yaeT |
| --- | --- | --- | --- |
| Arsenophonus nasoniae str. *α*Nv_FIN | GCF_004768525.1 | | |
| Arsenophonus apicola str. ArsBeeUS | GCF_020268605.1 | | |
| Arsenophonus apicola str. ArsBeeCH | GCF_903968575.1 | | |
| Arsenophonus nasoniae (DSM 15247) | AUCC00000000.1 | | |
| Arsenophonus of Polyomatus bellargus str. *α*Pb | OP205267 | OP205268 | OP205269 |
| Candidatus Arsenophonus melophagi | http://users.prf.jcu.cz/novake01/ | | |
| Arsenophonus of Nilaparvata lugens str. Hangzhou | JRLH00000000.1 | | |
| Arsenophonus of Aphis craccivora (isolate Ash) | NZ_CP038155.1 | | |
| Arsenophonus of Bemisia tabaci Asia II 3 | NZ_MASH00000000.1 | | |
| Arsenophonus endosymbiont of Muscidifurax uniraptor | GU226813.1 | GU226798.1 | GU226786.1 |
| Arsenophonus endosymbiont of Protocalliphora azurea | GU226815.1 | GU226801.1 | GU226787.1 |
| Arsenophonus endosymbiont of Pachycrepoideus vindemmiae | GU226812.1 | GU226799.1 | GU226784.1 |
| Arsenophonus endosymbiont of Spalangia cameroni | GU226814.1 | GU226800.1 | GU226785.1 |
| Arsenophonus nasoniae | GU226811.1 | GU226797.1 | GU226783.1 |
| Arsenophonus endosymbiont of Aphis gossypii | GU226822.1 | GU226808.1 | GU226795.1 |
| Arsenophonus endosymbiont of Aphis spiraecola | GU226823.1 | GU226809.1 | GU226796.1 |
| Arsenophonus endosymbiont of Cacopsylla alaterni | GU226821.1 | GU226807.1 | GU226794.1 |
| Arsenophonus endosymbiont of Bemisia tabaci | GU226816.1 | GU226805.1 | GU226788.1 |
| Arsenophonus endosymbiont of Trialeurodes vaporariorum | GU226820.1 | GU226806.1 | GU226793.1 |
| Arsenophonus endosymbiont of Triatoma guasayana | GU226818.1 | GU226803.1 | GU226789.1 |
| Arsenophonus endosymbiont of Triatoma infestans | GU226817.1 | GU226804.1 | GU226790.1 |
| Arsenophonus endosymbiont of Hippobosca equina | GU226819.1 | GU226810.1 | GU226791.1 |
| Proteus mirabilis | NC_010554.1 | | |
| Providencia stuartii | NC_017731.1 | | |
